# Improving the Accuracy of Direction of Arrival Estimation with Multiple Signal Inputs Using Deep Learning

**DOI:** 10.3390/s24102971

**Published:** 2024-05-07

**Authors:** Yihan Lu, Hengchao Guan, Kun Yang, Tong Peng, Chengyuan Wen, Xin Li

**Affiliations:** School of Information Engineering, Zhejiang Ocean University, Zhoushan 316022, China; luyihan@zjou.edu.cn (Y.L.); guanhengchao@zjou.edu.cn (H.G.); xin.li@zjou.edu.cn (X.L.)

**Keywords:** DOA estimates, CAPON, CNN, noise reduction

## Abstract

In this paper, an innovative cyclic noise reduction method and an improved CAPON algorithm (also the called minimum variance distortionless response (MVDR) algorithm) are proposed to improve the accuracy and reliability of DOA (direction of arrival) estimation. By processing the eigenvalues obtained from the covariance matrix of the received signal, the signal-to-noise ratio (SNR) can be increased by up to 5 dB by the cyclic noise reduction method, which will improve the DOA estimation accuracy. The improved CAPON algorithm has a convolution neural network (CNN) structure, whose input is the processed covariance matrix of the received signal, and the CAPON spectral value is used as the training label to obtain the estimated spatial spectrum. It retains the advantages of the CAPON algorithm, which can achieve blind source estimation, and simulations show that the proposed algorithm exhibits a better performance than the traditional algorithm in conditions of various SNRs and snapshot numbers. The simulation results show that, based on a certain SNR, the root mean square error (RMSE) of the improved CAPON algorithm can be reduced from 0.86° to 0.8° compared to traditional algorithms, and the angle estimation error can be decreased by up to about 0.3°. With the help of the cyclic noise reduction method, the angle estimation error decreases from 0.04° to 0.02°.

## 1. Introduction

DOA estimation is an application of array signal processing [1,2]. Usually, an array is composed of several sensors arranged in a certain form and distributed in different positions in space. All the elements in the array sense the spatial signals, which will be used to determine the direction of multiple target sources. Nowadays, DOA estimation is widely used in various fields such as radar antennas, sonar systems, and wireless network positioning. In radar antenna design, designers utilize this technology to optimize the layout of the antenna array and the configuration of antenna elements in order to more precisely determine the direction of signals [3]. By accurately controlling the directivity and beam width of the antenna, it is possible to effectively avoid or reduce interference from other signal sources, thus enhancing the radar’s detection capabilities towards targets [4].

Signal denoising is a traditional classical problem. The first denoising algorithms were used to smooth signals and eliminate singular points in receiving signals. Such denoising algorithms include the mean algorithm and median algorithm [5]. We find that eigenvalue decomposition of the covariance matrix can improve the SNR by about 5 dB after the minimum noise variance is repeatedly subtracted from the signal and noise characteristics. In this paper, we name this method ’cyclic noise reduction’, and it will be described in detail in later sections.

Traditional DOA estimation algorithms are model-based estimation methods, which determine the signal parameters through mathematical derivation and related super-resolution algorithms. However, these algorithms usually have a poor estimation performance under harsh conditions. Subspace-based DOA estimation methods, such as the multiple signal classification (MUSIC) algorithm [6], require complex operations such as matrix eigenvalue decomposition and spectral peak searching, and a lot of computing resources. Minimum variance spectral estimation (MVM), proposed by Capon [7] in 1969, is an adaptive beamforming method and is not constrained by the array aperture. However, the disadvantage with these algorithms is that when the SNR is low, the DOA estimation errors are large. In view of the above problems, some scholars have adopted machine learning techniques [8,9,10] such as neural networks and support vector machines [11] to estimate the DOA. Compared with traditional machine learning techniques, deep learning (DL) does not rely on interpretable model designs and can autonomously learn from a large amount of raw data to extract and mine features. DL models have a superior predictive performance, and once trained, the network requires only simple inference to complete estimation tasks [12,13]. Current DL-based DOA estimation studies typically use the received signals and their processed forms as network inputs, with the outputs being the corrected received signals or predicted angles [14,15]. Furthermore, DL has also been used to enhance traditional DOA estimation methods. For instance, Elbir [16] proposed the Deep MUSIC algorithm, which uses a learning-based data-driven architecture to learn the nonlinear relationship between input and output data through neural networks, achieving good DOA estimation results. However, the number of signal sources still needs to be preset.

Current DOA estimation methods based on deep learning can roughly be divided into two categories. In the first group of methods, the DOA estimation problem is transformed into a neural network classification problem. The spatial angles are classified and the mapping relationship between the network input data and the DOA is learnt. For example, Liu et al. [17] proposed a two-level framework, which uses a multi-task autoencoder to roughly divide the parameter space and a parallel multi-layer classifier to further divide the sub-region. The framework uses the real and imaginary parts of the array receiving data covariance as input, and then extracts features to achieve high-precision direction estimation in the presence of array errors. In the second group of methods, the DOA estimation problem is transformed into a neural network regression problem, and features related to location information are extracted from the received signal and directly mapped to the source location through deep learning. For example, Wu et al. [18] regarded DOA estimation as a sparse linear inverse problem in compressed sensing, proposing a deep convolutional network that learns the inverse transformation from a large training dataset using sparse priors to obtain the DOA of the signal effectively in real time.

In this paper, a regression model with ULA is used in the improved CAPON algorithm. By preprocessing the covariance of the real complex signal received by the uniform linear array, the DOA is estimated after training with the CAPON spectrum value as the label. The reduction in noise proves that, in the case of Gaussian noise, the algorithm combined with machine learning not only outputs better DOA estimation results in terms of accuracy, but also has more advantages in low-SNR environments compared to the traditional CAPON algorithm.

The remainder of this paper is structured as follows. Section 1 presents the data model of bearing estimation for uniform linear arrays. Section 2 describes the `cyclic noise reduction’ method. In Section 3, we present the network architecture. In Section 4, the simulations and performance evaluations are described. Finally, Section 5 concludes the paper.

## 2. Array Signal Model

**Figure 1:**
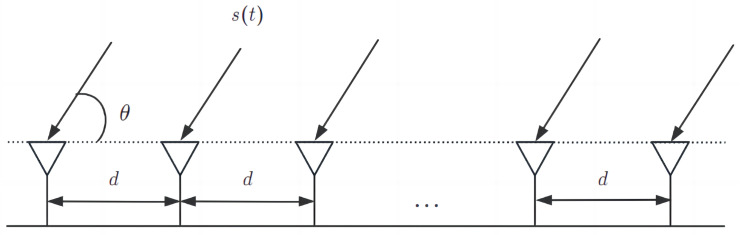
Uniform linear array.

Consider a uniform linear array, as shown in Figure 1. It is assumed that *K* far-field narrowband non-coherent target signal sources impinge on an *M*-element uniform linear array with an arbitrary distance *d* from the angle θ [19]. The received signal can be expressed as:

(1) $$X\left(t\right)=\underset{k=1}{\sum^{K}}{a\left(\theta_{k}\right)s}_{k}\left(t\right)+N\left(t\right)=A\left(\theta \right)S\left(t\right)+N\left(t\right)$$

where $s_{k}\left(t\right)$ denotes the *k*th source’s transmitting signals at time *t*, $N\left(t\right)$ is the Gaussian white additive noise with mean value 0 and variance $\sigma^{2}$, ${N\left(t\right)=\left[n_{1}\left(t\right),n_{2}\left(t\right),\ldots ,n_{M}\left(t\right)\right]}^{T}$, and ${\left[\cdot \right]}^{T}$ denotes the transpose operation.

(2) $${a\left(\theta_{k}\right)=\left[1,e^{-j2\pi d\lambda \sin\theta_{k}},\ldots ,e^{-j2\pi \left(M-1\right)d\lambda \sin\theta_{k}}\right]}^{T}$$

is the steering vector,

(3) $$A\left(\theta \right)=\left[a\left(\theta_{1}\right),a\left(\theta_{2}\right),\ldots ,a\left(\theta_{k}\right)\right]$$

is the steering matrix of the array, and λ is the wavelength of the signal. The covariance matrix of the array output X(t) is:

(4) $$R_{xx}=E\left[{X\left(t\right)X}^{H}\left(t\right)\right]={A\left(\theta \right)R}_{s}A^{H}\left(\theta \right)+\sigma^{2}I$$

where E[·] denotes mathematical expectation and ${\left[\cdot \right]}^{H}$ denotes the conjugate transpose operation. $R_{s}$ denotes the covariance matrix of the signal source and I is the identity matrix.

## 3. Noise Reduction Preprocessing

**Figure 2:**
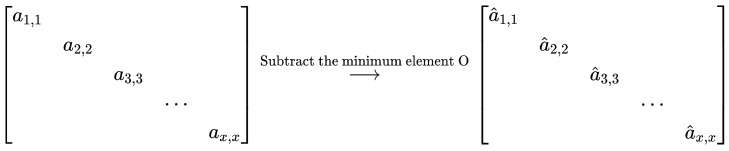
Noise reduction by subtracting the minimum value of the diagonal elements.

Based on the covariance matrix $R_{xx}$, we can compute the eigenvalues D (a diagonal matrix) and eigenvector V. Under the assumption that the number of array sensors is larger than the number of signal sources, the minimum value of D will be the noise variance $\sigma^{2}$. To reduce the noise impact, we subtract this minimum value from D to obtain $D_{n}$, as shown in Figure 2. Thus, the reduced noise covariance matrix is obtained by multiple $D_{n}$ and V, which can be used in the proposed CNN structure to estimate DOAs.

The accuracy of this process depends on the length of the received signal, as the limited number of samples can lead to errors in real situations. Therefore, to reduce the approximation error of the covariance matrix, a large number of samples need to be considered.

In order to verify the accuracy of the cyclic noise reduction method, we study environments with SNRs of 0 dB and 5 dB and actual angle values of −51.5°, −7°, and 8.85° using the MUSIC algorithm.

**Figure 3:**
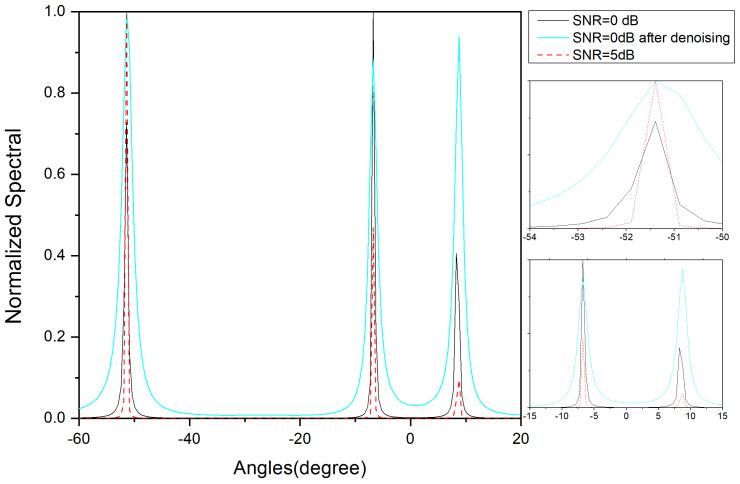
Normalized-power spectrum with SNR = 0 dB, SNR = 5 dB, and SNR = 0 dB after denoising.

From Figure 3, it can be observed that at an SNR of 0 dB, the MUSIC algorithm estimates the angles with a slight error. At an SNR of 5 dB, the MUSIC algorithm can estimate the angles more accurately. At an SNR of 0 dB, the algorithm employing the cyclic noise reduction method achieves an estimation performance that is almost equivalent to that of the algorithm without noise reduction at an SNR of 5 dB, which indicates that the cyclic noise reduction method results in an SNR improvement of roughly 5 dB.

## 4. Improved CAPON and CAPON

### 4.1. CAPON Algorithm and CAPON Algorithm Estimation Framework

The main advantage of the CAPON algorithm lies in its practicality, as it operates effectively without the need for prior knowledge or assumptions regarding signals. At the same time, the algorithm also has a certain anti-interference ability and robustness and can be applied to different types of signal processing with different complexities.

The CAPON algorithm analyzes signals and noise by collecting array signals and computing the covariance matrix. The core step involves optimizing the weight vector using the Lagrange multiplier method, with the goal of minimizing the output power of the received signals while ensuring that the gain in the desired signal direction remains unchanged. Following this optimization, the optimal weight vector *w* is obtained [7], as shown in Equation (5). In this equation, $R^{-1}$ represents the inverse of the signal covariance matrix, θ denotes the array response vector, and $a^{H}\left(\theta \right)$ is the conjugate transpose of a(θ). The optimal weight vector is:

(5) $$w=\frac{R^{-1}a\left(\theta \right)}{a^{H}\left(\theta \right)R^{-1}a\left(\theta \right)}$$

The power spectrum in the CAPON algorithm is the reciprocal of the output power of the beamformer in each direction. Convert the above optimal weight vector to:

(6) $$P\left(\theta \right)=\frac{1}{w^{H}R_{w}}=\frac{1}{a^{H}\left(\theta \right)R^{-1}a\left(\theta \right)}$$

By scanning θ, the corresponding CAPON power spectrum P(θ) is obtained. After the angle search, the maximum point of the spatial spectrum is found, which is the direction of arrival of the signal.

The resolution of the CAPON algorithm is limited by the array geometry and the signal wavelength, which means that it may be difficult to distinguish these signals when the angle of the incident signal is very low. At a low SNR, due to the weak signal strength, noise will have a significant impact on the estimation of the covariance matrix, which will reduce the effect of the weight vector and thus affect the DOA estimation accuracy. In order to solve this problem, we design an improved CAPON algorithm combined with machine learning. By establishing a nonlinear mapping between the signal and the useful parameters from the data, an estimated angle of arrival is obtained. By expanding the dataset and modifying the model, the algorithm has improved anti-noise abilities and a high resolution.

**Figure 4:**
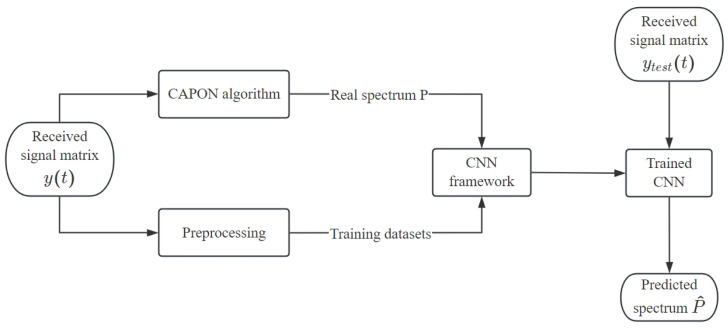
Improved CAPON algorithm framework.

The estimation framework of the improved CAPON algorithm is shown in Figure 4. Directly using the received signal as input to construct a neural network model will not only increase the complexity of the model’s structure, but also increase the difficulty of model convergence during training and significantly increase the size of the required training sample. Therefore, methods such as the time difference, phase difference, covariance matrix of the received signal, and eigenvector of the covariance matrix are usually used to preprocess the received data to reduce their dimensions and extract key eigenvalues.

### 4.2. Dataset Construction

**Figure 5:**
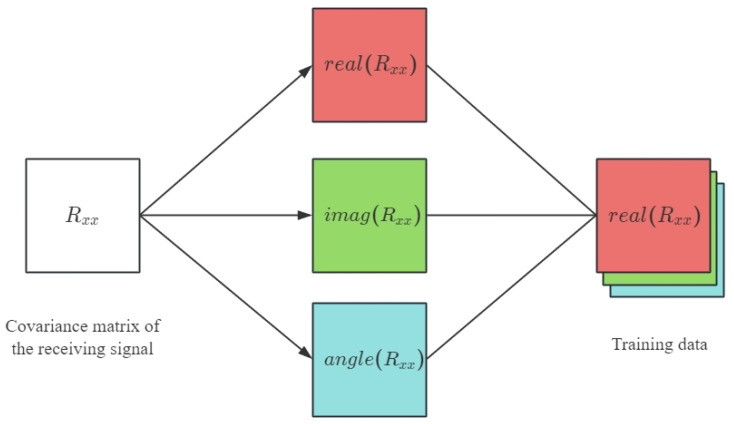
Transformation from the covariance matrix to training data.

In order to construct an input dataset for our proposed network framework, we used the real and imaginary parts and angles of the covariance matrix $R_{y}$. Let X be a real-valued matrix with a size of M×M×3; then, the (i,j)-th element of the first and second ’channel’ of X is given by ${\left[\left[X\right]:,:,1\right]}_{i,j}=Re{\left\{R_{xx}\right\}}_{i,j}$ and ${\left[\left[X\right]:,:,2\right]}_{i,j}=Im{\left\{R_{xx}\right\}}_{i,j}$, respectively. A schematic diagram of the construction of matrix X is illustrated in Figure 5. Similarly, the (i,j)-th element of the third ’channel’ of X is defined as ${\left[\left[X\right]:,:,3\right]}_{i,j}=∠{\left\{R_{xx}\right\}}_{i,j}$, where ∠· denotes the angle information of a complex number. The processed input information is sent to the neural network, and the real CAPON spectrum value is used as a label for training. After normalizing the output of the network, the corresponding angle value is selected by using a segmentation line with a threshold of 0.5.

For the improved CAPON algorithm model, the angle range is set to [−60°,60°]. Firstly, one angle is selected every 5° within the range of [−60°,60°], yielding a total of $N_{\theta }=25$ angle values. Subsequently, for each of these angles, k=100 reception signal matrices X are generated using the spatial channel model (SCM). To ensure improved detection at a higher SNR, SNR values were chosen at intervals of 5 dB within the range of [−10,10] dB, resulting in a total of $N_{SNR}=5$ SNR values. Gaussian white noise corresponding to these SNRs is added to the reception signal matrices, with noise for each SNR value being added $N_{noise}=1000$ times to mitigate random effects. Following this, the covariance matrix of the received signal $R_{xx}$ is computed to generate a training set $\bar{R}$, and the CAPON algorithm is utilized to calculate the spatial spectrum p corresponding to $R_{xx}$, which is used as the label set $\tilde{u}$ for training the network. The dataset $E_{S}$ for training has a size of $k\times N_{\theta }\times N_{SNR}\times N_{noise}=1,250,000$, with each dataset consisting of data pairs $\left(\bar{R},\tilde{u}\right)$. Prior to the initiation of training, the entire dataset is shuffled, and 80% of the dataset is allocated as the training set and the remainder is designated as the validation set.

### 4.3. Model Design and Training

**Figure 6:**
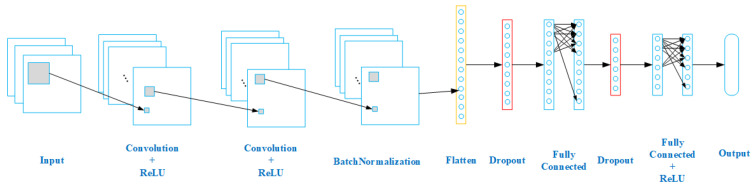
Architecture of the proposed CNN.

The network structure of the improved CAPON algorithm model is shown in Figure 6. This model is mainly responsible for reconstructing the input matrix and establishing a nonlinear relationship with the CAPON spectrum value. In this paper, the task is modeled as a regression task. The purpose is to fit the mapping relationship to the power spectrum value *P* and $R_{xx}$ according to Equation (4), which is defined as the nonlinear function from the input space to the output space, namely $f_{CNN}:R^{M\times M\times 3}\to R^{L}$. The nonlinear function $f_{CNN}$ is parameterized by an eight-layer CNN, where $f_{CNN}\left({\bar{R}}_{x}=f^{\left(8\right)}\left(f^{\left(7\right)}\left(\cdots f^{\left(1\right)}\left({\bar{R}}_{x}\right)\right)\right)\right)=\hat{u}$. Specifically, the function ${\left\{f^{\left(i\right)}\left(\cdot \right)\right\}}_{i=\left\{1,2,3\right\}}$ represents a series of convolutional layers: a two-dimensional convolutional layer containing 512 neurons with a convolution kernel size of 5×5, a Max-pooling layer, and a two-dimensional convolutional layer containing 512 neurons with a convolution kernel size of 3×3. $f^{\left(4\right)}\left(\cdot \right)$ is the batch normalization function, $f^{\left(5\right)}\left(\cdot \right)$ is the Flatten layer, which is used to convert the output of the batch normalization layer into a one-dimensional vector, $f^{\left(6\right)}\left(\cdot \right)$ is the Dropout layer, and the parameter is set to 0.3. $f^{\left(7\right)}\left(\cdot \right)$ is a fully connected layer containing 1024 neurons and the ReLU activation function. The final output layer $f^{\left(8\right)}\left(\cdot \right)$ is a fully connected layer containing floor (r/q) neurons, in which *r* is the angle range of scanning, while *q* is the number of partitions. The output of the neural network is expressed as $\hat{u}={\left[{\hat{u}}_{1},\cdots ,{\hat{u}}_{N}\right]}^{T}$.

In network training, the mean square error (MSE) between the actual output and the label is used as the loss function.

(7) $$L_{\mathrm{MSE}}\left({\tilde{u}}^{\left(k,i\right)},{\hat{u}}^{\left(k,i\right)}\right)=\frac{1}{N}\sum_{n=1}^{N}{\left({\tilde{u}}_{n}^{\left(k,i\right)}-{\hat{u}}_{n}^{\left(k,i\right)}\right)}^{2}$$

The optimizer used is Adam, the learning rate is 0.001, the number of hidden layers is 8, the batch size is 128, the number of training rounds is 100, and the training is terminated when the accuracy of the verification set no longer improves after five iterations.

## 5. Simulation Results and Analysis

In this section, the estimation performance of the proposed method is verified via simulations. The experimental software environment is TensorFlow on RTX 3090. The number of Monte Carlo experiments was set to $M_{c}=100$, the number of snapshots was set to T=200, and the receiving array was a ULA with an interval of d=0.15 m. Next, the performance of the CNN is demonstrated by evaluating its performance in DOA estimation. It should be noted that in order to avoid the influence of the data consistency between the test and training sets on the experimental results, the incident angle used in this experiment was added to the selected angle with a random value in the range of [−0.5,0.5]. In terms of DOA estimation, the performance of the improved CAPON in this chapter is compared with MUSIC. The root mean square error (RMSE) is used to evaluate the DOA estimation performance, which is defined as follows:

(8) $$RMSE=\sqrt{\frac{1}{KM_{C}}\sum_{k=1}^{K}\sum_{t=1}^{M_{C}}{\left(\theta_{k}-{\hat{\theta }}_{k,t}\right)}^{2}}$$

Here, the true value of the *k*-th DOA angle is $\theta_{k}$ and the estimated value of the *k*-th DOA angle in the *t*-th Monte Carlo experiment is ${\hat{\theta }}_{k,t}$.

### 5.1. Effect of the SNR on Estimation Accuracy

The relationship between the RMSEs of the angle and the SNRs of the CAPON, MUSIC, and the improved CAPON methods is shown in Figure 5.

**Figure 7:**
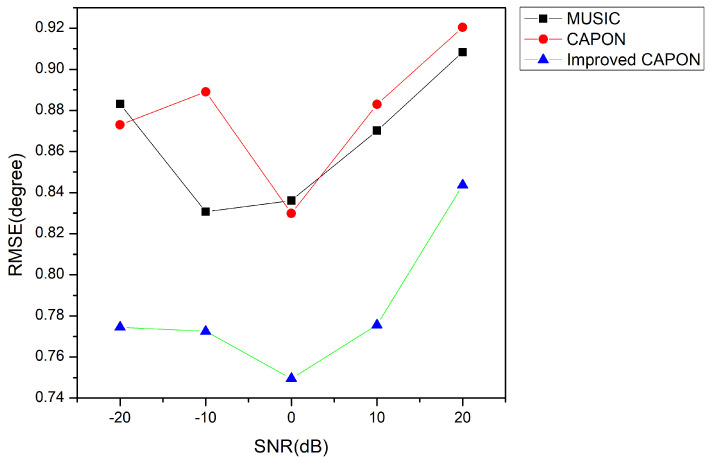
RMSE variation at different SNRs.

It can be seen from Figure 7 that the performance of the improved CAPON algorithm is significantly better than that of the traditional algorithm. The performance is best around an SNR of 0 dB, where the improved CAPON algorithm can fully learn the signal characteristics. Although it outperforms traditional algorithms in the range of 10 dB to 20 dB, the RMSE metrics remain comparatively elevated. This is because the signal characteristics are already quite apparent compared to the noise characteristics in this range, and finding angle information by minimizing the output power is challenging, which to some extent also affects the performance of the improved CAPON algorithm.

In the range of −10 dB to 10 dB, we studied the spectral value–angle plots at SNRs of −10, 5, and 10, as shown below.

**Figure 8:**
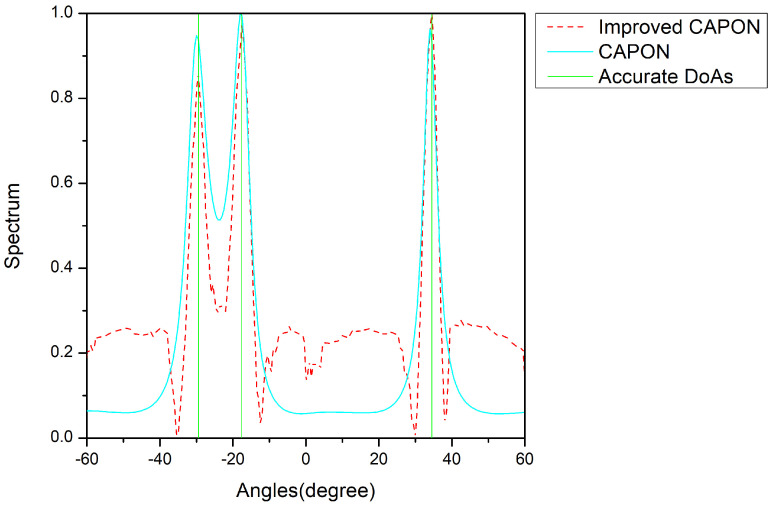
Normalized-spectrum at SNR = −10 dB; the accurate DOAs are −29.5°, −17.5°, and 34.5°.

**Figure 9:**
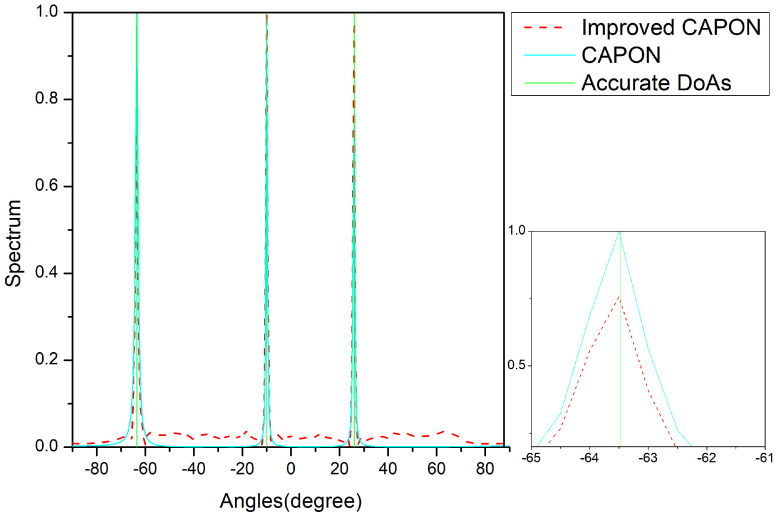
Normalized-spectrum at SNR = 5 dB; the accurate DOAs are −63.5°, −10°, and 25°.

**Figure 10:**
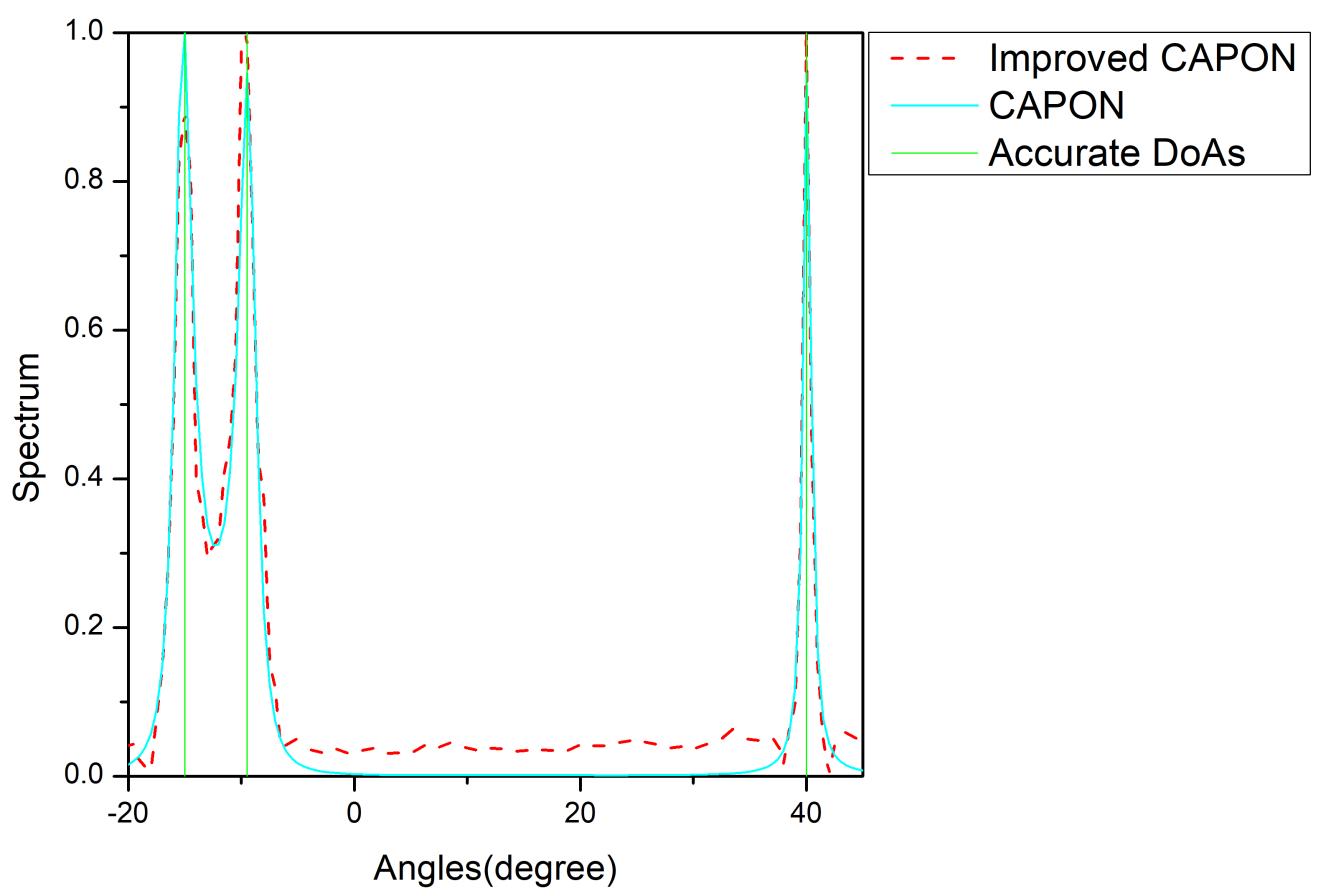
Normalized-spectrum at SNR = 10 dB; the accurate DOAs are −15°, −8°, and 40°.

In Figure 8, Figure 9 and Figure 10, the vertical lines are the true angle values, where the red lines indicate the spectral values normalized by the improved CAPON algorithm and the blue lines indicate the spectral values normalized by the CAPON algorithm. The spectral peaks of the improved CAPON algorithm are clearly closer to the vertical lines of the true angle values, i.e., the DOA values estimated by the improved CAPON algorithm deviate less from the true angles. Taking Figure 8 as an example, the true angle values are −29.5°, −17.5°, and 34.5° at an SNR of −10 dB, and the values estimated by the CAPON algorithm are −29.95°, −17.9°, and 34.05°, respectively. The improved CAPON algorithm estimates values of −29.62°, −17.69°, and 34.22°, respectively; thus, it can be seen that the angle estimates of the improved CAPON algorithm are more accurate. Compared to the CAPON algorithm, the improved CAPON algorithm’s actual angle error is reduced by about 0.3°, demonstrating a very accurate angular measurement estimation.

### 5.2. The Effect of Adding Noise Reduction

**Figure 11:**
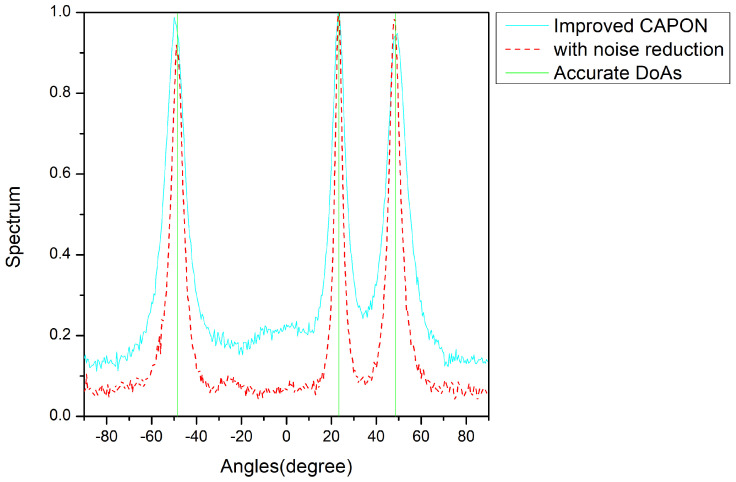
Normalized-spectrum comparisons using noise reduction; the accurate angles are −17.4°, −6.3°, and 27°.

After using the cyclic noise reduction method, simulations were performed with parameters consistent with those above, and the SNR was set to 0 dB. The estimation accuracy is shown in Figure 11.

From Figure 11, it can be seen that after noise reduction, the performance of the improved CAPON is improved at the same SNR. With the help of the noise reduction method, the angle estimation error decreases from 0.04° to 0.02°. The results of these comprehensive analyses show that the improved CAPON algorithm performs better in combination with the cyclic noise reduction algorithm.

### 5.3. Effect of the Snapshot Number on Estimation Accuracy

**Figure 12:**
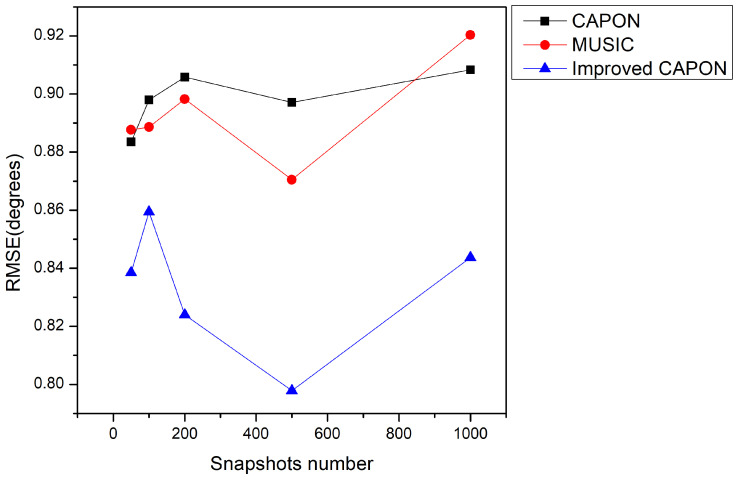
Differences in algorithm performance at varying snapshot lengths.

In this section, the impact of the number of snapshots on the proposed algorithm’s performance is analyzed. The structural parameters of the array are consistent with those used in the above sections. The SNR of the training and test sets is 5 dB. The number of snapshots used in the test was set between 50 and 1000. The root mean square error of angle measurements for different snapshot numbers is shown in Figure 12.

From Figure 12, it can be seen that at different snapshot counts, the performance of the improved CAPON algorithm is consistently better than that of the traditional algorithm. Particularly, in scenarios with a high number of snapshots, taking the case of 500 snapshots as an example, the RMSE of the improved CAPON algorithm decreases from 0.86° to 0.8°. This is because data with a high number of snapshots are more diverse in time or space, which can help the neural network extract features that are more complex and distinctive. If evaluated based on the number of snapshots, the improved CAPON algorithm is suitable for scenarios with multiple snapshots and exhibits a superior performance.

### 5.4. Summary of Simulation Results

The improved CAPON algorithm outperforms the traditional CAPON algorithm, as shown in Figure 7, Figure 8, Figure 9 and Figure 10, especially at SNR = 0 dB, where it learns signal traits better, leading to more precise DOA estimates. Figure 8 highlights that this algorithm has a reduced error by about 0.3°, indicating closer estimates to the true angles.

Figure 11 highlights the performance improvement after cyclic noise reduction, and Figure 12 showcases the improved results across different snapshot counts, notably for a high snapshot number. This is due to the algorithm’s ability to leverage diverse data for more detailed feature extraction.

The accuracy of the improved CAPON algorithm primarily stems from its proficient assimilation of signal characteristics, which is especially effective in environments with an abundance of snapshot data. The performance can be further enhanced by integrating the cyclic noise reduction algorithm.

## 6. Conclusions

In this paper, we propose an improved CAPON algorithm based on a neural network technique. The simulations show that the CNN-based method performs better in the mid- and low-SNR regions, and its results are superior to those of CAPON and MUSIC when the amount of snapshots is large. The DOA estimation method proposed in this paper has a higher positioning accuracy and a lower error rate than the traditional CAPON algorithm at a low SNR. In addition, the proposed method does require knowledge of the number of signal sources in advance and has good adaptability and robustness. The experimental results show that deep learning technology can effectively extract signal features, thereby improving the DOA estimation accuracy.

We describe a radio wave propagation environment with distant scattering clusters. Thus, the received signal is a linear combination of the signals from the effective scattering clusters. The DOA is the center of a scattering cluster, which is the key to building correlated MIMO channels. Adding up all the scattering clusters, we can reconstruct the physical propagation environment for radio waves.

## Data Availability

Data are contained within the article.
